# *IL28B*, *HLA-C*, and *KIR* Variants Additively Predict Response to Therapy in Chronic Hepatitis C Virus Infection in a European Cohort: A Cross-Sectional Study

**DOI:** 10.1371/journal.pmed.1001092

**Published:** 2011-09-13

**Authors:** Vijayaprakash Suppiah, Silvana Gaudieri, Nicola J. Armstrong, Kate S. O'Connor, Thomas Berg, Martin Weltman, Maria Lorena Abate, Ulrich Spengler, Margaret Bassendine, Gregory J. Dore, William L. Irving, Elizabeth Powell, Margaret Hellard, Stephen Riordan, Gail Matthews, David Sheridan, Jacob Nattermann, Antonina Smedile, Tobias Müller, Emma Hammond, David Dunn, Francesco Negro, Pierre-Yves Bochud, Simon Mallal, Golo Ahlenstiel, Graeme J. Stewart, Jacob George, David R. Booth, for the International Hepatitis C Genetics Consortium (IHCGC)

**Affiliations:** 1 Storr Liver Unit, Westmead Millennium Institute, University of Sydney, Sydney, Australia; 2 Institute for Immunology and Allergy Research, Westmead Millennium Institute, University of Sydney, Sydney, Australia; 3 Institute for Infectious Diseases, Murdoch University, Perth, Australia; 4 Cancer Research Program, Garvan Institute for Medical Research, University of New South Wales, Sydney, Australia; 5 School of Mathematics and Statistics, University of New South Wales, Sydney, Australia; 6 Medizinische Klinik m.S. Hepatologie und Gastroenterologie, Charité, Campus Virchow-Klinikum, Universitätsmedizin Berlin, Germany; 7 Department of Hepatology, Clinic for Gastroenterology and Rheumatology, University Clinic Leipzig, Leipzig, Germany; 8 Department of Gastroenterology and Hepatology, Nepean Hospital, Sydney, Australia; 9 Liver Physiopathology Lab, Department of Internal Medicine, University of Turin, Turin, Italy; 10 Department of Internal Medicine I, University of Bonn, Bonn, Germany; 11 Liver Research Group, Institute of Cellular Medicine, Medical School, Newcastle University, Newcastle upon Tyne, United Kingdom; 12 National Centre in HIV Epidemiology and Clinical Research, University of New South Wales, Sydney, Australia; 13 St Vincent's Hospital, Sydney, Australia; 14 NIHR Biomedical Research Unit in Gastroenterology and the Liver, University of Nottingham, Nottingham, United Kingdom; 15 Princess Alexandra Hospital, Department of Gastroenterology and Hepatology, Woolloongabba; 16 The University of Queensland, School of Medicine, Princess Alexandra Hospital, Woolloongabba, Queensland, Australia; 17 Burnet Institute, Commercial Road, Melbourne, Victoria, Australia; 18 Gastrointestinal and Liver Unit, Prince of Wales Hospital and University of New South Wales, Sydney, Australia; 19 Divisions of Gastroenterology, Hepatology, and Clinical Pathology, University Hospitals Geneva, Switzerland; University of Oxford, United Kingdom

## Abstract

**Background:**

To date, drug response genes have not proved as useful in clinical practice as was anticipated at the start of the genomic era. An exception is in the treatment of chronic hepatitis C virus (HCV) genotype 1 infection with pegylated interferon-alpha and ribavirin (PegIFN/R). Viral clearance is achieved in 40%–50% of patients. Interleukin 28B (*IL28B*) genotype predicts treatment-induced and spontaneous clearance. To improve the predictive value of this genotype, we studied the combined effect of variants of *IL28B* with human leukocyte antigen C (*HLA-C*), and its ligands the killer immunoglobulin-like receptors (*KIR*), which have previously been implicated in HCV viral control.

**Methods and Findings:**

We genotyped chronic hepatitis C (CHC) genotype 1 patients with PegIFN/R treatment-induced clearance (*n* = 417) and treatment failure (*n* = 493), and 234 individuals with spontaneous clearance, for *HLA-C* C1 versus C2, presence of inhibitory and activating *KIR* genes, and two *IL28B* SNPs, rs8099917 and rs12979860. All individuals were Europeans or of European descent. *IL28B* SNP rs8099917 “G” was associated with absence of treatment-induced clearance (odds ratio [OR] 2.19, *p* = 1.27×10^−8^, 1.67–2.88) and absence of spontaneous clearance (OR 3.83, *p* = 1.71×10^−14^, 2.67–5.48) of HCV, as was rs12979860, with slightly lower ORs. The *HLA-C* C2C2 genotype was also over-represented in patients who failed treatment (OR 1.52, *p* = 0.024, 1.05–2.20), but was not associated with spontaneous clearance. Prediction of treatment failure improved from 66% with *IL28B* to 80% using both genes in this cohort (OR 3.78, *p* = 8.83×10^−6^, 2.03–7.04). There was evidence that *KIR2DL3* and *KIR2DS2* carriage also altered HCV treatment response in combination with *HLA-C* and *IL28B*.

**Conclusions:**

Genotyping for *IL28B*, *HLA-C*, and *KIR* genes improves prediction of HCV treatment response. These findings support a role for natural killer (NK) cell activation in PegIFN/R treatment-induced clearance, partially mediated by *IL28B*.

*Please see later in the article for the Editors' Summary*

## Abstract 2

Vijayaprakash Suppiah and colleagues show that genotyping hepatitis C patients for the *IL28B*, *HLA-C*, and *KIR* genes improves the ability to predict whether or not patients will respond to antiviral treatment.

## Editors' Summary

**Background:**

About 170 million people harbor long-term (chronic) infections with the hepatitis C virus (HCV) and 3–4 million people are newly infected with the virus every year. HCV—a leading cause of chronic hepatitis (inflammation of the liver)—is spread though contact with infected blood. Transmission can occur during medical procedures (for example, transfusions with unscreened blood or reuse of inadequately sterilized medical instruments) but in developed countries, where donated blood is routinely screened for HCV, the most common transmission route is needle-sharing among intravenous drug users. HCV infection can cause a short-lived illness characterized by tiredness and jaundice (yellow skin and eyes) but 70%–80% of newly infected people progress to a symptom-free, chronic infection that can eventually cause liver cirrhosis (scarring) and liver cancer. HCV infections can be treated with a combination of two drugs—pegylated interferon-alpha and ribavirin (PegIFN/R). However, PegIFN/R is expensive, causes unpleasant side-effects, and is ineffective in about half of people infected with HCV genotype 1, the commonest HCV strain.

**Why Was This Study Done?:**

It would be extremely helpful to be able to identify which patients will respond to PegIFN/R before starting treatment. An individual's genetic make-up plays a key role in the safety and effectiveness of drugs. Thus, pharmacogenomics—the study of how genetic variants affects the body's response to drugs—has the potential to alter the clinical management of many diseases by allowing clinicians to provide individually tailored drug treatments. In 2009, scientists reported that certain single nucleotide polymorphisms (SNPs, a type of genetic variant) lying near the *IL28B* gene (which encodes an immune system protein made in response to viral infections) strongly influence treatment outcomes and spontaneous clearance in HCV-infected people. This discovery is now being used to predict treatment responses to PegIFN/R in clinical practice but genotyping (analysis of variants of) *IL28B* only correctly predicts treatment failure two-thirds of the time. Here, the researchers investigate whether genotyping two additional regions of the genome—the *HLA-C* and *KIR* gene loci—can improve the predictive value of *IL28B* genotyping. Human leukocyte antigen C (HLA-C) and the killer immunoglobulin-like receptors (KIRs) are interacting proteins that have been implicated in HCV viral control.

**What Did the Researchers Do and Find?:**

The researchers genotyped 417 patients chronically infected with HCV genotype 1 whose infection had been cleared by PegIFN/R treatment, 493 patients whose infection had not responded to treatment, and 234 patients whose infection had cleared spontaneously for two *HLA-C* variants (C1 and C2), the presence of several KIR genes (individuals carry different combinations of KIR genes), and two *IL28B* SNPs (rs8099917 and rs12979860). Carriage of “variants” of either *IL28B* SNP was associated with absence of treatment-induced clearance and absence of spontaneous clearance. That is, these variant SNPs were found more often in patients who did not respond to treatment than in those who did respond, and more often in patients who did not have spontaneous clearance of their infection than those who did. The HLA-C C2C2 genotype (there are two copies of most genes in the genome) was also more common in patients who failed treatment than in those who responded but was not associated with spontaneous clearance. The rate of correct prediction of treatment failure increased from 66% with *IL28B* genotyping alone to 80% with combined *IL28B* and *HLA-C* genotyping. Finally, carriage of specific *KIR* genes in combination with specific *HLA-C* and *IL28B* variants was also associated with an altered HCV treatment response.

**What Do These Findings Mean?:**

These findings show that the addition of *HCL-C* and *KIR* genotyping to *IL28B* genotyping improved the prediction of HCV treatment response in the patients investigated in this study. Because all these patients were European or of European descent, these findings need confirming in people of other ethnic backgrounds. They also need confirming in other groups of Europeans before being used in a clinical setting. However, the discovery that the addition of *HLA-C* genotyping to *IL28B* genotyping raises the rate of correct prediction of PegIFN/R treatment failure to 80% is extremely promising and should improve the clinical management of patients infected with HCV genotype 1. In addition, these results provide new insights into how PegIFN/R clears HCV infections that may lead to improved therapies in the future.

**Additional Information:**

Please access these websites via the online version of this summary at http://dx.doi.org/10.1371/journal.pmed.1001092.

- The World Health Organization provides detailed information about hepatitis C (in several languages)
- The US Centers for Disease Control and Prevention provides information on hepatitis C for the public and for health professionals (information is also available in Spanish)
- The US National Institute of Diabetes and Digestive and Kidney Diseases provides basic information on hepatitis C (in English and Spanish)
- The Hepatitis C Trust is a patient-led, patient-run UK charity that provides detailed information about hepatitis C and support for patients and their families; a selection of personal stories about patients' experiences with hepatitis C is available, including Phil's treatment story, which details the ups and downs of treatment with PegIFN/R
- MedlinePlus provides links to further resources on hepatitis C
- The Human Genome Project provides information about medicine and the new genetics, including a primer on pharmacogenomics

## Introduction

Studies of human genetics have been expected to alter clinical management for many diseases, including infectious diseases. Yet, to date, there are few examples of the use of such information in routine clinical practice. One of the most promising examples, identified in genome-wide analyses, is used to predict response to treatment for hepatitis C, based on a single genetic variant.

Only 20%–30% of the ∼170 million people infected with the hepatitis C virus (HCV) recover spontaneously; the remainder develop chronic infection [1] with a risk for developing cirrhosis, liver failure, and hepatocellular carcinoma [2]. Current standard of care with pegylated interferon-alpha and ribavirin (PegIFN/R) achieves a sustained virological response (SVR) (HCV RNA undetectable 6 mo post cessation of therapy) in 40%–50% of those infected with the most common viral genotype, type 1, after 48 wk [3]. Treatment is expensive and is associated with numerous side effects, which sometimes require dose reduction and premature treatment cessation, thus increasing the risk of treatment failure. Host genotyping studies have the potential to identify genes and therefore pathogenic processes important in viral clearance, enabling a rational approach to design new drugs, and to identify patients who will most likely respond to current and new treatments.

We and others previously used genome-wide association studies (GWAS) to identify SNPs in the genetic region encoding *IL28B*, which strongly influences treatment outcome [4]–[6] and spontaneous clearance [7]. Other genes associated with drug response have not yet been identified in GWAS with genome-wide significance. Variants in linkage with *IL28B* allow prediction of up to 64% for failure to clear virus during therapy in cross-sectional cohorts [5]. The minor allele of SNP rs8099917 tags the nonresponse haplotype in Asians and Caucasians, but not in African Americans. The minor allele of SNP rs12979860 is on this haplotype in all ethnic groups, and on other less significant haplotypes, so is used where African Americans are in the patient cohort [8].

GWAS are dependent on SNPs tagging associated genetic variants and cannot measure interactions because of the high statistical penalty for multiple comparisons. Human leukocyte antigen (HLA) and killer cell immunoglobulin-like receptors (KIR) are highly polymorphic genetic loci whose gene proteins interact with each other and for which proxy SNPs for their major variants have yet to be identified. HLA-C molecules present ligands for KIR2DL receptors, with a functionally relevant dimorphism determining KIR specificity: *HLA-C* group 1 (HLA-C1) alleles, identified by Ser77/Asp80 of the HLA-C alpha 1 domain, are ligands for the inhibitory receptors KIR2DL2 and KIR2DL3 and the activating receptor KIR2DS2 [9],[10]. *HLA-C* group 2 (*HLA-C2*) alleles, identified by Asp77/Lys80, are recognized by inhibitory KIR2DL1 and activating KIR2DS1 [9]–[12]. KIR2DL3 and its ligand, HLA-C1 has been associated with an increased likelihood of spontaneous [13]–[15], and treatment-induced HCV clearance [14],[15]. This association is attributed to differential natural killer (NK) cell activation and function in the context of this KIR/HLA interaction [16]. SNPs from the *HLA-C* coding regions showed weak associations with SVR in our original GWAS [5].

The current study specifically addresses whether the *IL28B* and *KIR/HLA-C* gene loci have separate, additive, or interactive effects on HCV clearance (spontaneous or treatment induced). This information is essential to better understand the role of *IL28B* during HCV infection, to better predict response to therapy, and potentially to allow better selection of patients for treatment.

## Methods

### Ethics Statement and Study Participants

Ethical approval was obtained from the Human Research Ethics Committees of Sydney West Area Health Service and the University of Sydney. All other sites had ethical approval from their respective ethics committees. Written informed consent was obtained from all participants. Characteristics of each cohort are shown in Table 1. All treated patients were infected with genotype 1, received PegIFN/R, and had virological response determined 6 mo after completion of therapy. The diagnosis of chronic hepatitis C (CHC) was based on appropriate serology and presence of HCV RNA. All SVRs and non-SVR cases received therapy for 48 wk except when HCV RNA was present with a <2 log drop in HCV RNA level after 12-wk therapy. Patients were excluded if they had been coinfected with either hepatitis B virus or HIV or if they were not of European descent.

**Table 1:** Demographic characteristics for chronic hepatitis C patients after therapy, and for those participants with spontaneous virus clearance of HCV included in this study.

Demographic Factors^a^	Australian Cohort (*n* = 312)		Berlin Cohort (*n* = 310)		Newcastle, UK Cohort (*n* = 69)		Bonn Cohort (*n* = 57)		Trent, UK Cohort (*n* = 48)		Turin Cohort (*n* = 114)		Total Cohort (*n* = 910)		Participants with spontaneous virus clearance (*n* = 234)
	SVR (*n* = 130)	NSVR (*n* = 182)	SVR (*n* = 150)	NSVR (*n* = 160)	SVR (*n* = 31)	NSVR (*n* = 38)	SVR (*n* = 26)	NSVR (*n* = 31)	SVR (*n* = 22)	NSVR (*n* = 26)	SVR (*n* = 58)	NSVR (*n* = 56)	SVR (*n* = 417)	NSVR (*n* = 493)	
Age (y)	40.0 (9.6)	44.5 (7.1)	41.0 (10.5)	46.7 (10.3)	38.2 (11.8)	46.0 (12.0)	44.7 (12.9)	50.8 (10.9)	39.8 (9.8)	45.7 (7.9)	43.3 (13.1)	45.1 (10.0)	40.9 (10.8)	45.7^b^ (9.3)	NA
Gender (%)															
Females	52 (40.0)	42^b^ (23.1)	79 (52.7)	69 (43.1)	9 (29.0)	10 (26.3)	11 (42.3)	11 (35.5)	6 (27.3)	5 (19.2)	28 (48.3)	19 (33.9)	185 (44.4)	156^b^ (31.6)	111 (47.4)
Males	78 (60.0)	140 (76.9)	71 (47.3)	91 (55.9)	22 (71.0)	28 (73.7)	15 (57.7)	20 (64.5)	16 (72.7)	21 (80.8)	30 (51.7)	37 (66.1)	232 (55.6)	337 (68.4)	123 (52.6)
BMI	26.9 (5.1)	27.4 (5.3)	25.1 (4.5)	25.9 (3.9)	23.7 (6.3)	26.2 (6.6)	25.4 (4.2)	27.3 (4.6)	26.9 (3.5)	25.0 (2.9)	24.0 (3.2)	24.5 (3.3)	25.5 (4.7)	26.3 (4.7)	NA
Viral load^c^	NS	NS	*p*<0.05	*p*<0.05	*p*<0.05	*p*<0.05	*p*<0.05	*p*<0.05	NS	NS	*p*<0.05	*p*<0.05	*p*<0.05	*p*<0.05	NA

a Unless otherwise specified, mean (SD) are presented.

b *p*<0.05 comparisons between responders (SVR) and NSVR based on the χ^2^ test.

c Comparisons between SVR and NSVR based on the Mann-Whitney test. Viral load was measured differently between cohorts, so the data are presented simply as a statistical comparison within cohorts.

NA, not available; NS, not significant.

Samples from individuals with spontaneous clearance were collected from Westmead Hospital in Sydney (*n* = 149), the Melbourne NETWORK study (*n* = 31) [17], the Australian ATACH study (*n* = 18) [18], and Rheinische Friedrich-Wilhelms-Universitaet, Bonn, Germany (*n* = 36). Spontaneous clearance was defined as HCV RNA negative and hepatitis C antibody positive without undergoing hepatitis C treatment.

### Genotyping

For *HLA-C*, samples were genotyped by multiplex PCR [19] to two-digit resolution. For samples from Turin and those participants with spontaneous virus clearance, *HLA-C* genotyping was by PCR and sequencing. All Australian samples were genotyped by multiplex PCR for *KIR2DL2* and *KIR2DL3* [20]. *KIR2DL2* and *KIR2DL3* in the remainder and in those participants with spontaneous virus clearance and *KIR2DS1* and *KIR2DS2* in all samples were genotyped by PCR using the protocol of Ashouri et al. [21]. *2DL1* was not included owing to the fact that it is very common (>90%), so we would have insufficient power to detect an association with its absence. The rs8099917 SNP was genotyped as previously reported [5]. The IL28B rs12979860 SNP was genotyped using a custom made Taqman genotyping kit. Further details are reported in Text S1.

### Statistical Analysis

The Mann-Whitney and chi-squared tests were used to analyze baseline covariates. A chi-squared test was used to examine differences in allele, carriage, and genotype frequencies between SVR versus non-SVR (NSVR), those participants with spontaneous virus clearance (SC) versus CHC (i.e., NSVR plus SVR), and viral clearance (SC plus SVR) versus NSVR. The relationships between HLA-C, IL28B, and the KIR loci were investigated using logistic regression for predicting failure of SVR. Significance of all models was assessed by likelihood ratio (LR) tests. Analysis was carried out in R (v2.12).

## Results

### IL28B Genotype and HCV Viral Clearance

We had previously shown that the *IL28B* rs8099917 G allele predicts failure to clear HCV on PegIFN/R therapy [5], in the CHC cohort now analysed here for *HLA-C* and *KIR* genotypes. Carriers of the G allele were under-represented in SC (odds ratio [OR] 0.26, *p* = 1.71×10^−14^, 2.67–5.48) (Tables 2 and S1). The G allele appears to have a dominant effect, with both heterozygotes (OR 3.42, *p* = 1.32×10^−11^, 2.36–4.96) and homozygotes (OR 3.25, *p* = 1.78×10^−2^, 1.16–9.10) being similarly more likely to fail to clear virus spontaneously. SNP rs8099917 G carriers were 19.7% of SC, 27.5% of healthy controls (HapMap CEU [Utah residents with Northern or Western European ancestry] data), 37.9% of SVR, and 57.3% of NSVR (Table S1). Overall, those who failed to clear the virus after therapy or without therapy (NSVR versus SVR and SC) were much less likely to have the rs8099917 TT genotype (OR 0.34, *p* = 1.44×10^−17^) (Table S1), with both heterozygotes (OR 2.59, *p* = 5.91×10^−14^) and homozygotes (OR 2.12, *p* = 7.54×10^−3^) for the G allele more likely to fail to clear virus.

**Table 2:** Association of *IL28B* rs8099917 and *HLA-C* genotypes with viral clearance on therapy and spontaneous clearance.

Cohort	IL28			HLA-C			IL28/HLA-C^a^	
	TT	TG	GG	C1C1	C1C2	C2C2	C1*TT	C2C2 G*
**SVR (** ***n*** ** = 398/390/389)** b	247 (62.0)	134 (33.7)	17 (4.3)	151 (38.7)	185 (47.4)	54 (13.8)	200 (51.4)	13 (3.3)
**NSVR (** ***n*** ** = 475/463/459)** b	203 (42.7)	239 (50.3)	33 (6.9)	180 (38.9)	192 (41.5)	91 (19.7)	160 (34.9)	53 (11.5)
***p*** **-Value**	**1.27×10^−8^**	**7.35×10^−7^**	0.090	1.0	0.084	**0.024**	**1.17×10^−6^**	**8.83×10^−6^**
**OR, 95% CI**	**0.46, 0.35–0.60**	**2.0, 1.52–2.63**				**1.52, 1.05–2.20**	**0.51, 0.38–0.67**	**3.78, 2.03–7.04**
**Participants with spontaneous virus clearance (** ***n*** ** = 218/228/212)** a	175 (80.3)	39 (17.9)	4 (1.8)	95 (41.7)	105 (46.1)	28 (12.3)	147 (69.3)	2 (0.9)
**CHC (** ***n*** ** = 873/853/848)** ^c^	450 (51.5)	373(42.7)	50(5.7)	331(38.8)	377(44.2)	145(17.0)	360 (42.5)	69 (6.5)
***p*** **-Value**	**1.71×10^−14^**	**1.32×10^−11^**	**0.018**	0.43	0.62	0.084	**2.40×10^−12^**	**1.27×10^−3^**
**OR, 95% CI**	**0.26, 0.18–0.37**	**3.42, 2.36–4.96**	**3.25, 1.16–9.10**				**0.33, 0.24–0.45**	**7.31, 1.78–30.06**

Since the HLA-C genotype effect appears to be recessive, and IL28B genotype effect dominant, the genotypes of maximal difference are shown. C1*, C1 carriers; G*, G carriers. Comparisons with *p*<0.05 are in bold.

a Table S2 shows the data for all genotypes.

b Three different *n* represent the three sets of genotyping results being compared in this table, respectively.

### HLA-C2C2 Predicts Poor Viral Clearance on Therapy

**Figure 1:**
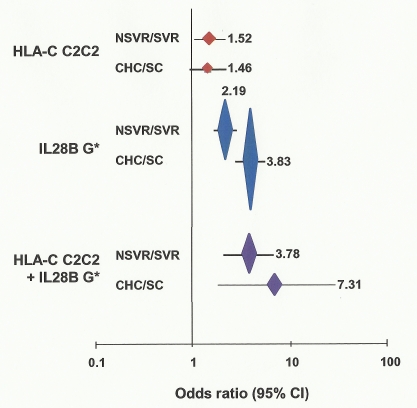
Association of HLA-C genotype with viral clearance with and without therapy. OR is plotted against viral clearance for each comparison, plus or minus 95% confidence interval (CI). Vertical height of plotted points is in proportion to log (1/p) where “*p*” is the probability of observed association being by chance. ORs are shown for each comparison. G*, carrier of G allele.

The *HLA-C1* and *C2* variants are associated with a number of aspects of viral clearance. *HLA-C2* homozygotes were more likely to fail to clear virus on therapy than other genotypes (OR 1.52, *p* = 0.025, 1.05–2.20) (Figure 1; Table 2). The HLA-C effect on viral clearance seems to be a recessive trait, such that *C1* heterozygotes are no more susceptible to treatment failure than *C1* homozygotes. *HLA-C2* homozygosity was not different between those participants with spontaneous virus clearance and healthy European controls (data for healthy controls from [22],[23]) (see Table S2). This important observation suggests that the difference in association of *HLA-C* genotype with viral clearance is due to response to therapy alone, not to the immune response in the absence of therapy. From two-digit genotyping of *HLA-C*, the *C2* variant conferring highest susceptibility to treatment failure is Cw*05 (OR 1.43, *p* = 0.047, 1.0–2.03) (Table S3). The Cw*03 variant of *C1* confers significant drug response (OR 0.61, *p* = 1.64×10^−3^, 0.44–0.83).

### Effect of *KIR* Genes on Viral Clearance

We tested if *KIR2DL2* or *2DL3* affected response to therapy, protection against development of CHC, or clearance of virus with or without PegIFN/R (Table S4). As reported by others [13],[14], we observed no effect of KIR genotype per se on viral clearance in any comparison. There was evidence of a similar trend between homozygosity of *HLA-C1* and *KIR2DL3* with SVR [14], and with spontaneous clearance [13]. Consistent with Knapp et al. [14] and Khakoo et al. [13], we found evidence that those infected with HCV and with the *KIR2DL3/C2C2* genotype, were more likely to fail to clear virus (OR 1.91, *p* = 0.022, 1.09–3.36) (Table S5) on therapy (NSVR versus SVR), and more common in those who failed to clear virus on therapy (NSVR) compared to those who did combined with those who cleared HCV without therapy (SVR+SC) (OR 2.08, *p* = 3.00×10^−3^, 1.27–3.40).

We next tested the combination of *HLA-C* alleles with *KIR2DL3* and *2DL2* genes (Table S6). There was evidence of increased association with the complementary pairs, so that the combination of the *C1* variant Cw*03 with its inhibiting genes was associated with increased treatment response: Cw*03 alone OR is 0.61 (*p* = 1.64×10^−3^, 0.44–0.83), with *2DL2* is 0.47 (*p* = 1.19×10^−3^, 0.29–0.75), with *2DL3* is 0.49 (*p* = 2.13×10^−4^, 0.33–0.72). Most of the *C2* association with treatment failure was due to allele Cw*05 (OR 1.43, *p* = 4.66×10^−2^, 1.0–2.03), with a larger effect in combination with the inactivating haplotype tagged by *2DL3* (OR 1.97, *p* = 2.04×10^−3^, 1.28–3.06), but unaffected by *2DL2*.

### Effect of Activating KIR Genes on Viral Clearance

The KIR ligands on NK cells activated on ligation to HLA-C are KIR2DS2 for HLA-C1, and KIR2DS1 for HLA-C2. Increased activation could occur in *HLA-C1* carriers who are also carriers of *KIR2DS2*, and for *HLA-C2* carriers who are also carriers of *KIR2DS1*. However, we found no evidence that *KIR2DS* genotypes affected viral clearance either singly or in combination with *HLA-C* genotypes (Tables S7 and S8), although from a logistic regression model, it seems that *KIR2DS1* could mitigate the effect of *HLA-C2C2* (see below).

### Combined Effect of HLA-C and IL28B Genotypes

**Figure 2:**
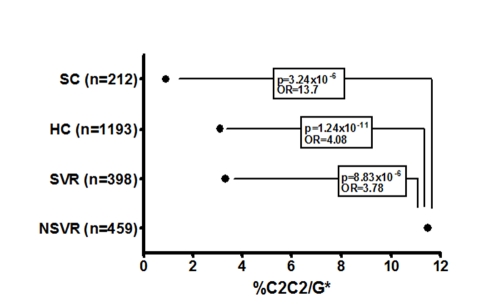
Proportion of each cohort with the HLA-C2C2 and IL28B G* genotype, which predicts treatment failure. HC, healthy controls; G*, carrier of G allele. Lines connect the significant 2×2 chi-squared comparisons with associated *p*-values and ORs. HC numbers obtained from Williams et al. [23] and Dunne et al. [22].

Prediction of failure to clear HCV in response to treatment with either *IL28B* or *HLA-C* genotypes alone is of limited value clinically due to the relatively low positive predictive value (PPV) for treatment failure [24]. We therefore tested if both genotypes together provided additional power to predict response. Indeed, the combination significantly improves prediction of failure to clear virus on therapy (OR 3.78, *p* = 8.83×10^−6^, 2.03–7.04), failure to clear virus spontaneously (OR 7.31, *p* = 1.27×10^−3^, 1.78–30.06), and failure to clear virus with and without therapy (OR 5.10, *p* = 2.53×10^−9^, 2.84–9.17) (Tables 2 and S9a). The largest difference was between those participants with spontaneous virus clearance and those who failed to clear virus on therapy (Figure 2). As shown in Table 3, prediction of treatment failure improved from 66% for *IL28B* G to 80% with *IL28B* G*/*C2C2*.

**Table 3:** Prediction of failure to clear virus on therapy with PegIFN/R.

Genotype	Sensitivity	Specificity	Positive Predictive Value	Negative Predictive Value
**IL28GG**	7	96	66	46
IL28G*	57	62	64	55
**HLA-C2C2**	20	86	63	47
HLA C2*	61	39	54	46
IL28G*/HLA-C2C2	12	97	**80** [Table-fn nt108]	**48** [Table-fn nt108]

Positive predictive value and negative predictive value for best genotype are in bold.

A similar predictive value for treatment response has been reported for *IL28B* SNP rs12979860^[4] ^We found that the PPV derived from combining this SNP and *HLA-C2C2* was actually lower in this cohort than for the rs8099917 combination (OR 2.52, *p* = 5.18×10^−5^, 1.59–3.98) (Table S9b). Adding the clinical features of age, gender, body mass index, or viral load improved predictive value (Figure S1, responder operator curves).

### Interactions between IL28B, HLA-C, and KIR

Using a logistic regression model, the increased OR of 3.78 for the combination rs8099917, G*/C2C2 is partially due to genetic interaction (LR, *p*<0.05) (Table S10), and not just an additive effect. Examining the relationship between *KIR* genotype and either *HLA-C2C2* or *IL28B* rs8099917 G*, we found no evidence for any two-way interactions for predicting failure of SVR. For the three-way interaction model between HLA-C, IL28, and not 2DS1, although the coefficient for the three-way interaction is significant, the LR test concludes that the model does not produce a significantly better fit (LR, *p* = 0.28). Although the HLA-C main effect is not significant via a standard *t*-test in the interaction model, it is associated with response (Table 2). Adding HLA-C to a model including rs8099917 leads to significant improvement of the fit (LR, *p* = 0.006), implying that HLA-C has an independent affect on response and should be included in the model. Adding the interaction term again improves the model fit (LR, *p* = 0.03).

## Discussion

The IL28B genotype is already used to predict treatment response to PegIFN/R in clinical practice, even though its association with therapeutic response was only first identified in late 2009. We tested the *IL28B*, *HLA-C*, and *KIR* gene variant associations with treatment-induced and spontaneous clearance of HCV and confirmed that *IL28B* rs8099917 predicts clearance in both situations. The *HLA-C2C2* genotype predicted failure to clear HCV on treatment, but no association with failure to clear HCV without treatment was detected. The prediction of treatment-induced clearance was additive and interactive between *IL28B* and *HLA-C*; and there was evidence of additive and interactive effects between *KIR2DL3*, *KIR2DS1*, and *HLA-C2C2*. These data and previous reports point to *HLA-C* as being the second gene predicting PegIFN/R treatment response in HCV. This genetic evidence supports an underlying physiological mechanism for HCV viral control involving an interaction between *IL28B*, *HLA-C*, and *KIR*s.

Khakoo et al. [13] and Dring et al. [25] compared *HLA-C* and *KIR* genotypes between those participants with spontaneous virus clearance and CHC, and Knapp et al. [14] in these and in treatment response. As in our study, Khakoo et al. reported *HLA-C2* homozygotes were more common in CHC than SC (OR 1.49, *p* = 0.02), and *KIR2DL3-C2* homozygotes slightly more so (OR 1.87, *p* = 0.01); and Dring et al. reported KIR2DS3-C2 carriers were more common in CHC than SC (OR 2.26, *p* = 0.002). Knapp et al. detected a trend towards C2 excess in those who failed to clear virus spontaneously compared to CHC (OR 1.69, *p* = 0.10), a trend of C2 excess in NSVR versus SVR (OR 1.38, *p* = 0.27) [13], but no difference between SVR and SC. In both the Knapp study and ours, the *KIR2DL3-C1* homozygotes were more common in SVR, and most of this association was due to the C1-Cw*03 variant. *KIR2DL3* tags haplotype A, which contains fewer activating *KIR* genes. This association is consistent with insufficient activation of NK cells in the context of HLA-C2C2 inhibition as the basis for increased risk of treatment failure.

Dring et al. [25] identified a dramatic synergy between KIR2DS3 (not examined here, encoded on haplotype B) and the IL28B SNP rs12979860 in predicting spontaneous clearance in a unusually homogenous cohort of Irish females infected with genotype 1 HCV by transfusion. They also showed that IFNλ inhibited IFNγ production by NK cells. They did not examine SNP rs8099917 or SVR and NSVR. Their data further support NK function in HCV clearance as being influenced by IFNλ.

Because of the very high linkage disequilibrium in the MHC class I region around *HLA-C*, the association we and others have observed may be due to *HLA-C* variants tagging other class I genes. However, the KIR interactions, which are HLA-C specific, support the signal being due to *HLA-C* itself, as does the strong body of evidence pointing to the importance of NK cells in killing virally infected cells in response to interferon and tumor necrosis factor-alpha-related apoptosis-inducing ligand (TRAIL) (reviewed in [26]–[28]). In addition, activated NK cells recognize and lyse HCV replicon-containing hepatoma cells in vitro [27] and should therefore be able to kill virus-infected hepatocytes in vivo. Cells that lack or have downregulated MHC class I molecules, such as virally infected cells or tumour cells, are susceptible to NK cell-mediated killing. In this context it has been reported that IFNλ3 (the protein encoded by *IL28B*) augments the antitumor activity of NK cells [11],[27].

The association of HLA-C with viral clearance on treatment but not spontaneous clearance suggests that, on therapy, NK killing of hepatocytes is augmented in *HLA-C1* carriers compared to C2 homozygotes. There are numerous potential mechanisms by which *HLA-C* genotypes could affect NK cell activity in the context of IFNα treatment. IFNα could affect NK killing of HCV-infected hepatocytes. IFNα is known to increase NK sensitivity to activation [27], but also to directly activate NK cells in patients with HCV infection and induced a strongly cytotoxic phenotype [27]. This NK cell activation and killing of hepatocytes is affected by the *HLA-C* genotype: the C1 allele allowing activation more rapidly and aggressively [16]. *HLA-C* may be even more upregulated in response to IFNα [29], making it more difficult for C2 homozygotes to activate NK cells.

The association of *IL28B* genotype with SC and therapeutic response indicates that IFNλ3 affects viral clearance. IFNλ3 is likely to enhance antiviral mechanisms through upregulation of interferon-stimulated genes (ISGs) in acute disease [24], but its effect may be more complicated in chronic infection, in which upregulation of ISGs in liver is associated with reduced treatment response [30]. One approach to identifying the molecular pathways through which a genetic variant affects disease outcome is to identify other genes that affect pathogenesis, and especially those with which it may interact. The additive association of *HLA-C* and *IL28B* genotypes with treatment-induced clearance, but not spontaneous clearance, suggests *IL28B* may be enhancing NK killing on PegIFN/R therapy. *HLA-C* is one of the most upregulated genes following treatment of a cell line with IFNλ3 [29]. The degree of this upregulation may depend on *HLA-C* genotype.

The larger predictive value for HLA-C2C2 and IL28B rs8099917 G* than SNP rs12979860 T* may indicate different haplotype effects. There are five common *IL28B* haplotypes in Caucasians [5]. The rs8099917 minor allele tags the haplotype with the highest association with therapeutic response, whilst rs12979860 minor alleles are on this haplotype and others (Table S11). Ge et al. [4] reported that there was evidence of independent effects of the two haplotypes. Therefore the additive effect of *HLA-C* with *IL28B* may be only with the rs8099917-tagged haplotype. It is likely that these two SNPs, which were on genotyping chips, will be supplanted by others when a more comprehensive analysis of the genetic variation of IL28B is available.

The overall differences in frequency of *HLA-C2C2/IL28B* G* in healthy controls, those participants with spontaneous virus clearance, SVR, and NSVR groups suggests a role in pathogenesis for this gene combination. It is also striking that *HLA-C2C2* frequency is highly variable between ethnic groups, roughly in proportion to their treatment responsiveness (Figure S2). Much of the variation between African Americans and European Americans has been explained by the *IL28B* rs12979860 SNP [4], but it seems likely that the higher proportion of the *HLA-C2C2* genotype in African Americans may also contribute to their reduced viral clearance.

With regard to patient management, avoiding treatment in those less likely to respond to PegIFN/R is important given the toxicity of this treatment and the likelihood that one or multiple direct acting antiviral agents will soon be available [31],[32]. In this context, *IL28B* genotype alone allows prediction of failure of PegIFN/R in only 66% of patients (Table 3). We have shown that with *HLA-C* genotyping this can be improved to a clinically more meaningful 80%. Genotyping of *IL28B* and *HLA-C* to C1/C2 is rapid and inexpensive. Further genetic associations, including those affecting HLA-C/IFNλ interactions, viral sequence variability [33], and host/virus interactions such as IP-10 levels [34] might further enhance prediction of treatment outcomes.

In addition to supporting the importance of *HLA-C*, *KIR*, and *IL28B* in HCV clearance and drug response, and emphasizing the role of NK cells in the outcomes of HCV infection, this study highlights the value of investigating variants other than SNPs to identify genetic variants causing disease and drug response. Notably, independent replication of these data in Europeans, and testing them for the first time in African-Americans and other ethnic groups is required.

## Supporting Information

Figure S1 Responder operator curves for prediction of failure to clear virus on therapy based on clinical and genotyping data.
(DOC)

Figure S2 Proportion of each ethnic group with the genotype that predicts treatment failure: *HLA-C2C2* homozygotes and *IL28B* G carriers.
(DOC)

Table S1 Association of *IL28B* rs8099917 genotypes with viral clearance with and without therapy.
(DOC)

Table S2 Comparison of *HLA-C* group 1 and 2 allele and genotype distribution from previous studies.
(DOC)

Table S3 *HLA-C* (two-digit genotyping) in SVR and NSVR.
(DOC)

Table S4 Association of HLA-C inhibitory receptor genes *KIR2DL2* and *KIR2DL3* on viral clearance with and without therapy.
(DOC)

Table S5 Association of HLA-C inhibitory receptor genes *KIR2DL2* and *KIR2DL3* on viral clearance with and without therapy in combination with *HLA-C* genotypes.
(DOC)

Table S6 Association of HLA-C inhibitory receptor genes *KIR2DL2* and *KIR2DL3* on viral clearance in combination with *HLA-C* genotypes based on two-digit genotyping.
(DOC)

Table S7 Association of HLA-C activating receptor genes *KIR2DS1* and *KIR2DS2* on viral clearance with and without therapy.
(DOC)

Table S8 Association of HLA-C activating receptor genes *KIR2DS1* and *KIR2DS2* on viral clearance with and without therapy in combination with *HLA-C* genotypes.
(DOC)

Table S9 (**a**) Association of combinations of *IL28B* SNP rs8099917 and *HLA-C* genotypes on viral clearance with and without therapy. (**b**) Association of combinations of *IL28B* SNP rs12979860 and *HLA-C* genotypes on viral clearance with therapy.
(DOC)

Table S10 Odds ratios and corresponding *p*-values for predicting failure of SVR using logistic regression models.
(DOC)

Table S11 The distribution of the six common IL28B haplotypes bound by SNPs rs12980275 and rs8099917.
(DOC)

Text S1 Supplementary methods.
(DOC)

## Abbreviations

CHC: chronic hepatitis C
GWAS: genome-wide association study
HCV: hepatitis C virus
*HLA-C*: human leukocyte antigen C
*IL28B*: interleukin 28B
*KIR*: killer immunoglobulin-like receptors
LR: likelihood ratio
NK: natural killer
NSVR: no sustained viral response
OR: odds ratio
PegIFN/R: pegylated interferon-alpha and ribavirin
SC: spontaneous clearer
SVR: sustained viral response
