# Supplementary material for: IL28B, HLA-C, and KIR Variants Additively Predict Response to Therapy in Chronic Hepatitis C Virus Infection in a European Cohort: A Cross-Sectional Study
Source: PLoS Med. 2011 Sep 13;8(9):e1001092. doi: 10.1371/journal.pmed.1001092 (PMC3172251; doi:10.1371/journal.pmed.1001092)
Supplement: Figure S1 — Responder operator curves for prediction of failure to clear virus on therapy based on clinical and genotyping data. (DOC) [file pmed.1001092.s001.doc]

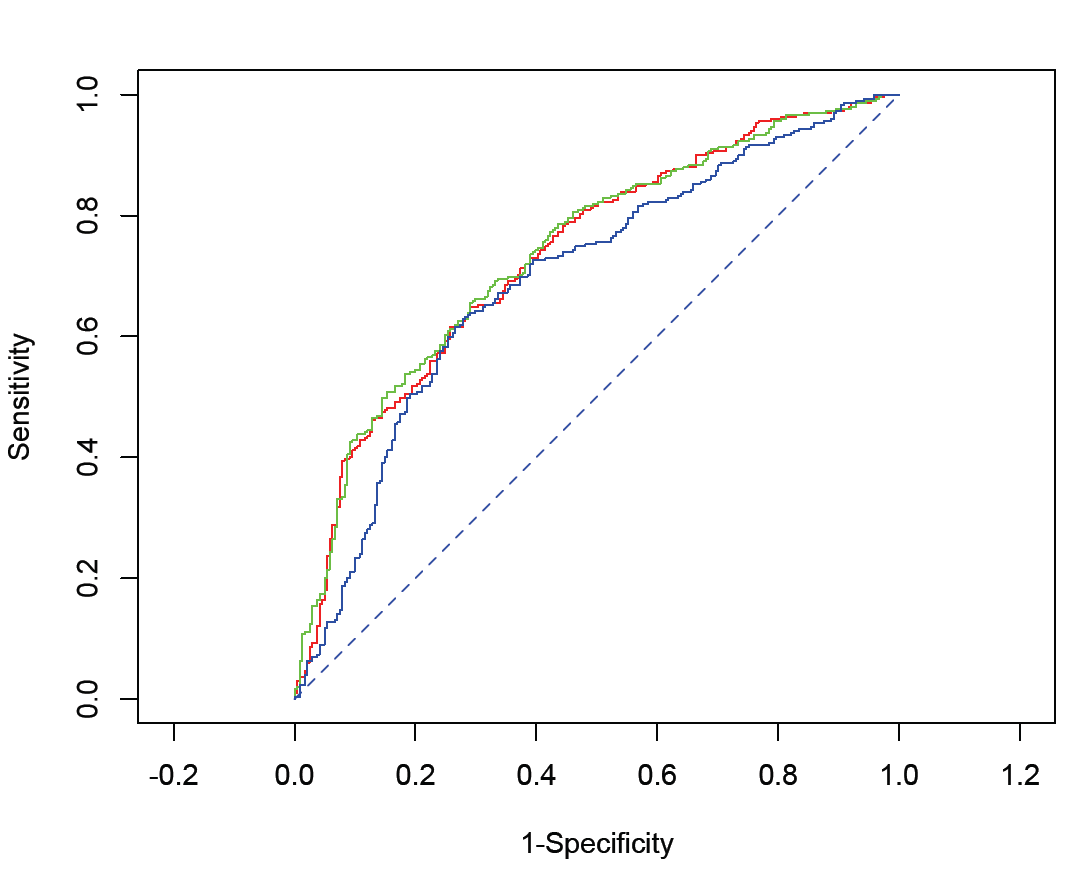


**Figure S1.** Receiver Operator Curves for prediction of failure to clear virus on therapy based on (blue line) clinical data only (age/sex/bmi/log(viral load)): AUC 69.5% ; (red line) Clinical + rs8099917: AUC 73.4% , (green line) Clinical + rs8099917*HLA.C: AUC 73.9% .
