# Supplementary material for: IL28B, HLA-C, and KIR Variants Additively Predict Response to Therapy in Chronic Hepatitis C Virus Infection in a European Cohort: A Cross-Sectional Study
Source: PLoS Med. 2011 Sep 13;8(9):e1001092. doi: 10.1371/journal.pmed.1001092 (PMC3172251; doi:10.1371/journal.pmed.1001092)
Supplement: Figure S2 — Proportion of each ethnic group with the genotype that predicts treatment failure: HLA-C2C2 homozygotes and IL28B G carriers. (DOC) [file pmed.1001092.s002.doc]

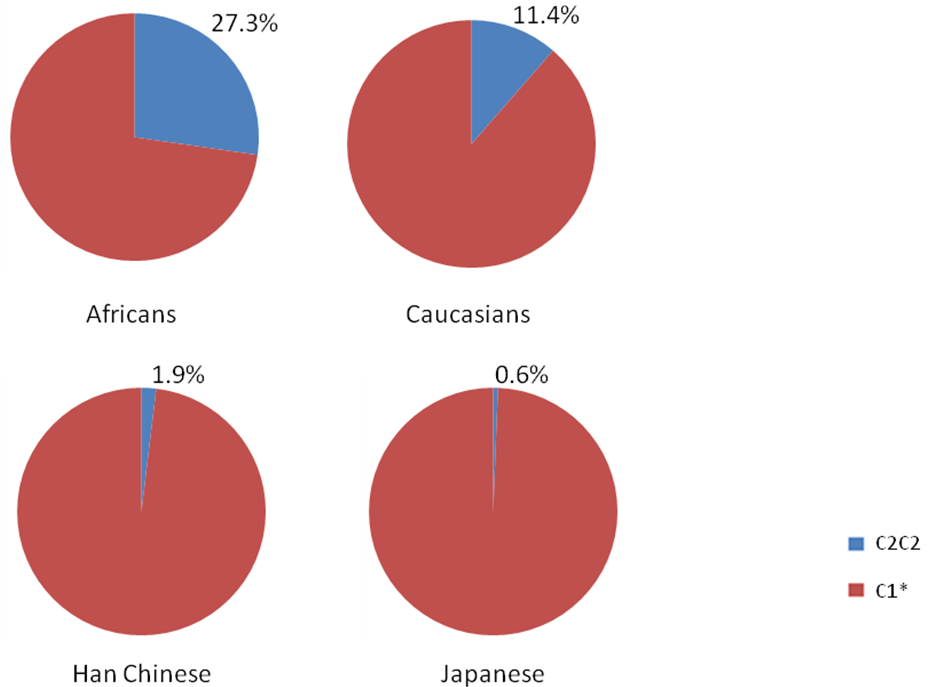


**Figure S2.** Proportion of each ethnic group with the *HLA-C* genotype which predicts treatment failure (blue).
