# Supplementary material for: IL28B, HLA-C, and KIR Variants Additively Predict Response to Therapy in Chronic Hepatitis C Virus Infection in a European Cohort: A Cross-Sectional Study
Source: PLoS Med. 2011 Sep 13;8(9):e1001092. doi: 10.1371/journal.pmed.1001092 (PMC3172251; doi:10.1371/journal.pmed.1001092)
Supplement: Table S1 — Association of IL28B rs8099917 genotypes with viral clearance with and without therapy. (DOC) [file pmed.1001092.s003.doc]

**Table S1.** Association of *IL28B* rs8099917 genotypes with viral clearance on with and without therapy

| **IL28B rs8099917**  **Genotype** | **Sustained Viral Response**  **(n=398)** | **No Sustained Viral Response**  **(n=475)** | **p value** | **OR, 95% CI** |
| --- | --- | --- | --- | --- |
| **T allele** | 628 (78.9) | 645 (67.9) | **2.59 x 10^-7^** | **0.57, 0.45-0.70** |
| **G allele** | 168 (21.1) | 305 (32.1) | **2.59 x 10^-7^** | **1.77, 1.42-2.20** |
| **T carriage** | 381 (95.7) | 442 (93.1) | 0.09 |  |
| **G carriage** | 151 (37.9) | 272 (57.3) | **1.27 x 10^-8^** | **2.19, 1.67-2.88** |
|  | **Spontaneous Clearers**  **(n=218)** | **Chronic Hepatitis C (n=873)** |  |  |
| **T allele** | 389 (89.2) | 1273 (72.9) | **8.64 x 10^-13^** | **0.33, 0.24-0.45** |
| **G allele** | 47 (10.8) | 473 (27.1) | **8.64 x 10^-13^** | **3.08, 2.23-4.24** |
| **T carriage** | 214 (98.2) | 823 (94.3) | **1.78 x 10^-2^** | **0.31, 0.11-0.86** |
| **G carriage** | 43 (19.7) | 423 (48.5) | **1.71 x 10^-14^** | **3.83, 2.67-5.48** |
|  | **No Sustained Viral Response**  **(n=475)** | **Spontaneous Clearers and SVR**  **(n=616)** |  |  |
| **T allele** | 645 (67.9) | 1017 (82.5) | **1.65 x 10^-15^** | **0.45, 0.37-0.55** |
| **G allele** | 305 (32.1) | 215 (17.5) | **1.65 x 10^-15^** | **2.24, 1.83-2.73** |
| **T carriage** | 442 (93.1) | 595 (96.6) | **7.54 x 10^-3^** | **0.47, 0.27-0.83** |
| **G carriage** | 272 (57.3) | 194 (31.5) | **1.44 x 10^-17^** | **2.91, 2.27-3.74** |
|  |  |  |  |  |
|  |  |  |  |  |
