# Supplementary material for: IL28B, HLA-C, and KIR Variants Additively Predict Response to Therapy in Chronic Hepatitis C Virus Infection in a European Cohort: A Cross-Sectional Study
Source: PLoS Med. 2011 Sep 13;8(9):e1001092. doi: 10.1371/journal.pmed.1001092 (PMC3172251; doi:10.1371/journal.pmed.1001092)
Supplement: Table S2 — Comparison of HLA-C group 1 and 2 allele and genotype distribution from previous studies. (DOC) [file pmed.1001092.s004.doc]

**Table S2.** Comparison of *HLA-C* Group 1 and 2 allele and genotype distribution from previous studies.

Figures are %. C1* and C2* denote carriers.

References are detailed in the manuscript. Samples from references 12 and 13 are predominantly of northern European origin. Reference 12 includes African Americans, in whom the C2 allele is significantly more common. Reference 13 cohorts are > 90% Caucasians. The healthy control data is for Irish samples, identified as clustered with Australian and American northern European origin samples^23^.

|  | **HC^22,23^** | **SC** | **SVR** | **NSVR** | **SC^12^** | **SC^13^** | **SVR^13^** | **NSVR^13^** |
| --- | --- | --- | --- | --- | --- | --- | --- | --- |
| **C1C1** | 43.6 | 41.7 | 38.7 | 38.9 | 37.5 |  |  |  |
| **C1C2** | 44.9 | 46.1 | 47.4 | 41.5 | 48.0 |  |  |  |
| **C2C2** | 11.6 | 12.3 | 13.8 | 19.7 | 14.5 |  |  |  |
|  |  |  |  |  |  |  |  |  |
| **C1*** | 88.4 | 87.8 | 86.1 | 80.4 | 85.5 | 83.3 | 87.9 | 75.2 |
| **C2*** | 56.4 | 58.4 | 61.2 | 61.2 | 62.5 | 58.3 | 58.9 | 66.3 |
|  |  |  |  |  |  |  |  |  |
