# Supplementary material for: IL28B, HLA-C, and KIR Variants Additively Predict Response to Therapy in Chronic Hepatitis C Virus Infection in a European Cohort: A Cross-Sectional Study
Source: PLoS Med. 2011 Sep 13;8(9):e1001092. doi: 10.1371/journal.pmed.1001092 (PMC3172251; doi:10.1371/journal.pmed.1001092)
Supplement: Table S3 — HLA-C (two-digit genotyping) in SVR and NSVR. (DOC) [file pmed.1001092.s005.doc]

**Table S3.** *HLA-C* (2 digit genotyping) in SVR and NSVR

| **HLA-C** | **SVR (%)**  **N=358** | **NSVR (%) N=426** | **p values^a^** | **OR (95% CI)^b^** |
| --- | --- | --- | --- | --- |
| **C1 Group** |  |  |  |  |
| **Cw*01** | 17 (2.4) | 12 (1.4) | 0.16 |  |
| **Cw*03** | 102 (14.2) | 78 (9.2) | **1.64 x 10^-3^** | **0.61, 0.44 – 0.83** |
| **Cw*07** | 220 (30.7) | 290 (34.0) | 0.16 |  |
| **Cw*08** | 24 (3.4) | 26 (3.1) | 0.74 |  |
| **Cw*12** | 42 (5.9) | 57 (6.7) | 0.50 |  |
| **Cw*14** | 9 (1.3) | 12 (1.4) | 0.79 |  |
| **Cw*16** | 37 (5.2) | 35 (4.1) | 0.32 |  |
|  |  |  |  |  |
| **C2 Group** |  |  |  |  |
| **Cw*02** | 35 (4.9) | 47 (5.5) | 0.58 |  |
| **Cw*04** | 80 (11.2) | 94 (11.0) | 0.92 |  |
| **Cw*05** | 54 (7.5) | 89 (10.4) | **0.047** | **1.43, 1.0 – 2.03** |
| **Cw*06** | 68 (9.5) | 76 (8.9) | 0.69 |  |
| **Cw*15** | 23 (3.2) | 25 (2.9) | 0.75 |  |
| **Cw*17** | 5 (0.7) | 11 (1.3) | 0.25 |  |
|  |  |  |  |  |

^a^P values were calculated by using chi-square test from 2x2 contingency tables; ^b^OR < 1 indicates a protective association with response to treatment
