# Supplementary material for: IL28B, HLA-C, and KIR Variants Additively Predict Response to Therapy in Chronic Hepatitis C Virus Infection in a European Cohort: A Cross-Sectional Study
Source: PLoS Med. 2011 Sep 13;8(9):e1001092. doi: 10.1371/journal.pmed.1001092 (PMC3172251; doi:10.1371/journal.pmed.1001092)
Supplement: Table S4 — Association of HLA-C inhibitory receptor genes KIR2DL2 and KIR2DL3 on viral clearance with and without therapy. (DOC) [file pmed.1001092.s006.doc]

**Table S4.** Association of HLA-C Inhibitory receptor genes *KIR2DL2* and *KIR2DL3* on viral clearance with and without therapy

| **KIR Genotype** | **Sustained Viral Response**  **(n=370)** | **No Sustained Viral Response**  **(n=441)** | **P value** |
| --- | --- | --- | --- |
| **2DL2** | 52 (14.1) | 58 (13.2) | 0.71 |
| **2DL3** | 157 (42.4) | 196 (44.4) | 0.57 |
| **2DL2 + 2DL3** | 146 (39.5) | 163 (37.0) | 0.47 |
|  |  |  |  |
|  | **Spontaneous Clearers**  **(n=234)** | **Chronic Hepatitis C**  **(n=811)** |  |
| **2DL2** | 38 (16.2) | 110 (13.6) | 0.30 |
| **2DL3** | 93 (39.7) | 353 (43.5) | 0.30 |
| **2DL2 + 2DL3** | 96 (32.5) | 309 (38.1) | 0.42 |
|  |  |  |  |
|  | **Viral Clearers**  **(n=604)** | **Viral Non-clearers**  **(n=441)** |  |
| **2DL2** | 90 (14.9) | 58 (13.2) | 0.42 |
| **2DL3** | 250 (41.4) | 196 (44.4) | 0.32 |
| **2DL2 + 2DL3** | 242 (40.1) | 163 (37.0) | 0.31 |
