# Supplementary material for: IL28B, HLA-C, and KIR Variants Additively Predict Response to Therapy in Chronic Hepatitis C Virus Infection in a European Cohort: A Cross-Sectional Study
Source: PLoS Med. 2011 Sep 13;8(9):e1001092. doi: 10.1371/journal.pmed.1001092 (PMC3172251; doi:10.1371/journal.pmed.1001092)
Supplement: Table S5 — Association of HLA-C inhibitory receptor genes KIR2DL2 and KIR2DL3 on viral clearance with and without therapy in combination with HLA-C genotypes. (DOC) [file pmed.1001092.s007.doc]

**Table S5.** Association of HLA-C Inhibitory receptor genes *KIR2DL2* and *KIR2DL3* on viral clearance with and without therapy in combination with *HLA-C* genotypes

| **HLA-C** | **KIR** | **Sustained Viral Response (n=359)** | **No Sustained**  **Viral Response(n=425)** | **P value** | **OR** |
| --- | --- | --- | --- | --- | --- |
| **C1-C1** | 2DL2 | 23 (6.4) | 18 (4.2) | 0.17 |  |
|  | 2DL3 | 64 (17.8) | 70 (16.5) | 0.62 |  |
|  | 2DL2 +2DL3 | 49 (13.6) | 64 (15.1) | 0.58 |  |
| **C1-C2** | 2DL2 | 25 (7.0) | 28 (6.6) | 0.84 |  |
|  | 2DL3 | 71 (19.8) | 78 (18.4) | 0.61 |  |
|  | 2DL2 +2DL3 | 67 (18.7) | 62 (14.6) | 0.13 |  |
| **C2-C2** | 2DL2 | 1 (0.3) | 9 (2.1) | - |  |
|  | 2DL3 | 19 (5.3) | 41 (9.6) | **0.022** | **1.91, 1.09-3.36** |
|  | 2DL2 +2DL3 | 27 (7.5) | 31 (7.3) | 0.92 |  |
|  |  |  |  |  |  |
|  |  | **Spontaneous Clearers**  **(n=234)** | **Chronic**  **Hepatitis C (n=784)** |  |  |
| **C1-C1** | 2DL2 | 14 (6.0) | 41 (5.2) | 0.65 |  |
|  | 2DL3 | 39 (16.7) | 134 (17.1) | 0.89 |  |
|  | 2DL2 +2DL3 | 31 (13.2) | 113 (14.4) | 0.65 |  |
| **C1-C2** | 2DL2 | 19 (8.1) | 53 (6.8) | 0.48 |  |
|  | 2DL3 | 43 (18.4) | 149 (19) | 0.82 |  |
|  | 2DL2 +2DL3 | 32 (13.7) | 129 (16.5) | 0.31 |  |
| **C2-C2** | 2DL2 | 4 (1.7) | 10 (1.3) | - |  |
|  | 2DL3 | 10 (4.3) | 60 (7.7) | 0.07 |  |
|  | 2DL2 +2DL3 | 10 (4.3) | 58 (7.4) | 0.09 |  |
|  |  |  |  |  |  |
|  |  | **Viral Clearers (n=593)** | **Viral Non-clearers (n=425)** |  |  |
| **C1-C1** | 2DL2 | 37 (6.2) | 18 (4.2) | 0.16 |  |
|  | 2DL3 | 103 (17.4) | 70 (16.5) | 0.71 |  |
|  | 2DL2 +2DL3 | 80 (13.5) | 64 (15.1) | 0.48 |  |
| **C1-C2** | 2DL2 | 44 (7.4) | 28 (6.6) | 0.61 |  |
|  | 2DL3 | 114 (19.2) | 78 (18.4) | 0.73 |  |
|  | 2DL2 +2DL3 | 99 (16.7) | 62 (14.6) | 0.36 |  |
| **C2-C2** | 2DL2 | 5 (0.8) | 9 (2.1) | 0.08 |  |
|  | 2DL3 | 29 (4.9) | 41 (9.6) | **3.1 x 10^-3^** | **2.08, 1.27-3.40** |
|  | 2DL2 +2DL3 | 37 (6.2) | 31 (7.3) | 0.51 |  |
