# Supplementary material for: IL28B, HLA-C, and KIR Variants Additively Predict Response to Therapy in Chronic Hepatitis C Virus Infection in a European Cohort: A Cross-Sectional Study
Source: PLoS Med. 2011 Sep 13;8(9):e1001092. doi: 10.1371/journal.pmed.1001092 (PMC3172251; doi:10.1371/journal.pmed.1001092)
Supplement: Table S6 — Association of HLA-C inhibitory receptor genes KIR2DL2 and KIR2DL3 on viral clearance in combination with HLA-C genotypes based on two-digit genotyping. (DOC) [file pmed.1001092.s008.doc]

**Table S6.** Association of HLA-C Inhibitory receptor genes *KIR2DL2* and *KIR2DL3* on viral clearance in combination with *HLA-C* genotypes based on 2 digit genotyping.

| **HLA-C^a^** | **Responders (%)**  **N=312** | **Non-responders (%) N=375** | **P values^b^** | **OR, 95% CI** |
| --- | --- | --- | --- | --- |
| **2DL2 +** |  |  |  |  |
| **C1 Group** |  |  |  |  |
| **Cw*01** | 8 (2.6) | 4 (1.1) | 0.14 |  |
| **Cw*03** | 52 (16.7) | 32 (8.5) | **1.19 x 10^-3^** | **0.47, 0.29-0.75** |
| **Cw*07** | 94 (30.1) | 129 (34.4) | 0.23 |  |
| **Cw*08** | 14 (4.5) | 12 (3.2) | 0.38 |  |
| **Cw*12** | 26 (8.3) | 28 (7.5) | 0.67 |  |
| **Cw*14** | 3 (1.0) | 5 (1.3) | - |  |
| **Cw*16** | 13 (4.2) | 15 (4.0) | 0.92 |  |
| **C2 Group** |  |  |  |  |
| ***Cw*02*** | 13 (4.2) | 21 (5.6) | 0.39 |  |
| ***Cw*04*** | 44 (14.1) | 41 (10.9) | 0.21 |  |
| ***Cw*05*** | 24 (7.7) | 42 (11.2) | 0.12 |  |
| ***Cw*06*** | 32 (10.3) | 32 (8.5) | 0.44 |  |
| ***Cw*15*** | 15 (4.8) | 10 (2.7) | 0.14 |  |
| ***Cw*17*** | 2 (0.6) | 5 (1.3) | - |  |
|  |  |  |  |  |
| **2DL3 +** |  |  |  |  |
| **C1 Group** |  |  |  |  |
| **Cw*01** | 14 (4.5) | 11 (2.9) | 0.28 |  |
| **Cw*03** | 80 (25.6) | 54 (14.4) | **2.13 x 10^-4^** | **0.49, 0.33-0.72** |
| **Cw*07** | 165 (52.9) | 212 (56.5) | 0.34 |  |
| **Cw*08** | 18 (5.8) | 23 (6.1) | 0.84 |  |
| **Cw*12** | 35 (11.2) | 42 (11.2) | 1 |  |
| **Cw*14** | 8 (2.6) | 11 (2.9) | 0.76 |  |
| **Cw*16** | 26 (8.3) | 25 (6.7) | 0.41 |  |
| **C2 Group** |  |  |  |  |
| ***Cw*02*** | 27 (8.7) | 35 (9.3) | 0.75 |  |
| ***Cw*04*** | 59 (18.9) | 70 (18.7) | 0.92 |  |
| ***Cw*05*** | 34 (10.9) | 73 (19.5) | **2.04 x 10^-3^** | **1.97, 1.28-3.06** |
| ***Cw*06*** | 48 (15.4) | 61 (16.3) | 0.75 |  |
| ***Cw*15*** | 19 (6.1) | 19 (5.1) | 0.56 |  |
| ***Cw*17*** | 3 (1.0) | 6 (1.6) | - |  |

^a^ *HLA-C* group 1 allotypes are shown in normal print and group 2 allotypes are shown in italics, ^b^P values were calculated by using chi-square test from 2x2 contingency tables
