# Supplementary material for: IL28B, HLA-C, and KIR Variants Additively Predict Response to Therapy in Chronic Hepatitis C Virus Infection in a European Cohort: A Cross-Sectional Study
Source: PLoS Med. 2011 Sep 13;8(9):e1001092. doi: 10.1371/journal.pmed.1001092 (PMC3172251; doi:10.1371/journal.pmed.1001092)
Supplement: Table S7 — Association of HLA-C activating receptor genes KIR2DS1 and KIR2DS2 on viral clearance with and without therapy. (DOC) [file pmed.1001092.s009.doc]

**Table S7.** Association of *HLA-C* Activating receptor genes *KIR2DS1* and *KIR2DS2* on viral clearance with and without therapy

| **KIR Genotype** | **Sustained Viral Response**  **(n=370)** | **No Sustained Viral Response**  **(n=441)** | **P value** |
| --- | --- | --- | --- |
| **2DS1** | 135 (36.5) | 177 (40.1) | 0.29 |
| **2DS2** | 181 (48.9) | 202 (45.8) | 0.38 |
|  |  |  |  |
|  | **Spontaneous Clearers**  **(n=234 2DS1/228 2DS2)** | **Chronic Hepatitis C**  **(n=811)** |  |
| **2DS1** | 108 (46.2) | 312 (38.5) | **0.035 0.73, 0.54-0.98** |
| **2DS2** | 115 (50.4) | 383 (47.2) | 0.39 |
|  |  |  |  |
|  | **Viral Clearers**  **(n=604/598)** | **Viral Non-clearers**  **(n=441)** |  |
| **2DS1** | 243 (40.2) | 177 (40.1) | 1 |
| **2DS2** | 296 (49.5) | 202 (45.8) | 0.24 |
