# Supplementary material for: IL28B, HLA-C, and KIR Variants Additively Predict Response to Therapy in Chronic Hepatitis C Virus Infection in a European Cohort: A Cross-Sectional Study
Source: PLoS Med. 2011 Sep 13;8(9):e1001092. doi: 10.1371/journal.pmed.1001092 (PMC3172251; doi:10.1371/journal.pmed.1001092)
Supplement: Table S8 — Association of HLA-C activating receptor genes KIR2DS1 and KIR2DS2 on viral clearance with and without therapy in combination with HLA-C genotypes. (DOC) [file pmed.1001092.s010.doc]

**Table S8.** Association of HLA-C activating receptor genes *KIR2DS1* and *KIR2DS2* on viral clearance with and without therapy in combination with *HLA-C* genotypes

| **HLA-C** | **KIR** | **Sustained Viral Response**  **(n=359)** | **No Sustained Viral Responses**  **(n=425)** | **P value** |
| --- | --- | --- | --- | --- |
| **C1-C1** | 2DS1 | 47 (13.1) | 61 (14.4) | 0.61 |
| **C1-C2** | 2DS1 | 67 (18.7) | 70 (16.5) | 0.42 |
| **C2-C2** | 2DS1 | 17 (4.7) | 37 (8.7) | **0.03 1.92, 1.06-3.47** |
|  |  |  |  |  |
| **C1-C1** | 2DS2 | 62 (17.3) | 71 (16.7) | 0.84 |
| **C1-C2** | 2DS2 | 89 (24.8) | 81 (19.1) |  |
| **C2-C2** | 2DS2 | 25 (7.0) | 41 (9.6) |  |
|  |  |  |  |  |
|  |  | **Spontaneous Clearers**  **(n=228 -DS1)** | **Chronic Hepatitis C**  **(n=784)** | **P value** |
| **C1-C1** | 2DS1 | 38 (16.7) | 108 (13.8) | 0.27 |
| **C1-C2** | 2DS1 | 53 (23.2) | 137 (17.5) | 0.05 |
| **C2-C2** | 2DS1 | 15 (6.6) | 54 (6.9) | 0.86 |
|  |  | (n=222 DS2) |  |  |
| **C1-C1** | 2DS2 | 44 (19.8) | 133 (17.0) | 0.32 |
| **C1-C2** | 2DS2 | 49 (22.1) | 170 (21.7) | 0.89 |
| **C2-C2** | 2DS2 | 18 (8.1) | 66 (8.4) | 0.89 |
|  |  |  |  |  |
|  |  | **Viral clearers (n=587-DS1)** | **Viral non-clearers (n=425)** | **P value** |
| **C1-C1** | 2DS1 | 85 (14.5) | 61 (14.4) | 1 |
| **C1-C2** | 2DS1 | 120 (20.4) | 70 (16.5) | 0.11 |
| **C2-C2** | 2DS1 | 32 (5.5) | 37 (8.7) | **0.04 1.65, 1.01-2.70** |
|  |  | **(n=581-DS2)** |  |  |
| **C1-C1** | 2DS2 | 106 (18.2) | 71 (16.7) | 0.53 |
| **C1-C2** | 2DS2 | 138 (23.8) | 81 (19.1) | 0.08 |
| **C2-C2** | 2DS2 | 43 (7.3) | 41 (9.6) | 0.20 |
