# Supplementary material for: IL28B, HLA-C, and KIR Variants Additively Predict Response to Therapy in Chronic Hepatitis C Virus Infection in a European Cohort: A Cross-Sectional Study
Source: PLoS Med. 2011 Sep 13;8(9):e1001092. doi: 10.1371/journal.pmed.1001092 (PMC3172251; doi:10.1371/journal.pmed.1001092)
Supplement: Table S9 — (a) Association of combinations of IL28B SNP rs8099917 and HLA-C genotypes on viral clearance with and without therapy. (b) Association of combinations of IL28B SNP rs12979860 and HLA-C genotypes on viral clearance with therapy. (DOC) [file pmed.1001092.s011.doc]

**Table S9a. Association of Combinations of *IL28B* SNP rs8099917 and *HLA-C* genotypes on viral clearance with and without therapy**

| **HLA-C Genotype** | **IL28B**  **rs8099917**  **Genotype** | **Sustained Viral Response**  **(n=389)** | **No Sustained Viral Response**  **(n=459)** | **P value** | **OR, 95% CI** |
| --- | --- | --- | --- | --- | --- |
| **C1-C1** | TT | 92 (23.7) | 77 (16.8) | **0.013** | **0.65, 0.46-0.91** |
|  | TG | 55 (14.1) | 92 (20.0) | **0.024** | **1.52, 1.06-2.19** |
|  | GG | 4 (1.0) | 11 (2.4) | 0.13 |  |
| **C1-C2** | TT | 108 (27.8) | 83 (18.1) | **7.71 x 10^-4^** | **0.57, 0.41-0.80** |
|  | TG | 65 (16.7) | 90 (19.6) | 0.28 |  |
|  | GG | 12 (3.1) | 16 (3.5) | 0.74 |  |
| **C2-C2** | TT | 40 (10.3) | 37 (8.1) | 0.26 |  |
|  | TG | 12 (3.1) | 47 (10.2) | **4.50 x 10^-5^** | **3.58, 1.87-6.86** |
|  | GG | 1 (0.3) | 6 (1.3) | - |  |
|  |  |  |  |  |  |
|  |  | **Spontaneous Clearers**  **(n=212)** | **Chronic**  **Hepatitis C**  **(n=1060)** |  |  |
| **C1-C1** | TT | 71 (33.5) | 240(22.6) | **7.92 x 10^-4^** | **0.58, 0.42-0.80** |
|  | TG | 17 (8.0) | 164(15.4) | **4.58 x 10^-3^** | **2.10, 1.24-3.54** |
|  | GG | 2 (0.9) | 17(1.6) | - |  |
| **C1-C2** | TT | 76 (35.8) | 267(25.1) | **1.41 x 10^-3^** | **0.60, 0.44-0.82** |
|  | TG | 17 (8.0) | 172(16.2) | **2.16 x 10^-3^** | **2.22, 1.32-3.74** |
|  | GG | 2 (0.9) | 30(2.8) | 0.11 |  |
| **C2-C2** | TT | 25 (11.8) | 102(9.6) | 0.33 |  |
|  | TG | 2 (0.9) | 61(5.7) | **3.20 x 10^-3^** | **6.41, 1.56-26.43** |
|  | GG | 0 | 7(0.6) | **-** |  |
|  | G* | 2 (0.9) | 69 (6) | **1.27 x 10^-3^** | **7.31, 1.78-30.06** |
|  |  |  |  |  |  |
|  |  | **Viral Clearers (n=601)** | **Viral non-clearers (n=459)** |  |  |
| **C1-C1** | TT | 163 (27.1) | 77 (16.8) | **6.68 x 10^-5^** | **0.54, 0.40-0.73** |
|  | TG | 72 (12.0) | 92 (20.0) | **3.22 x 10^-4^** | **1.84, 1.31-2.58** |
|  | GG | 6 (1.0) | 11 (2.4) | 0.073 |  |
| **C1-C2** | TT | 184 (30.6) | 83 (18.1) | **3.20 x 10^-6^** | **0.50, 0.37-0.67** |
|  | TG | 82 (13.6) | 90 (19.6) | **9.07 x 10^-3^** | **1.54, 1.11-2.14** |
|  | GG | 14 (2.3) | 16 (3.5) | 0.26 |  |
| **C2-C2** | TT | 65 (10.8) | 37 (8.1) | 0.13 |  |
|  | TG | 14 (2.3) | 47 (10.2) | **4.28 x 10^-8^** | **4.78, 2.60-8.80** |
|  | GG | 1 (0.2) | 6 (1.3) | **-** |  |
|  | G* | 15 | 53 | **2.53 x 10^-9^** | **5.10, 2.84-9.17** |

**Table S9b.** Association of Combinations of *IL28B* SNP rs12979860 and *HLA-C* genotypes on viral clearance with therapy

| **HLA-C Genotype** | **IL28B**  **Rs12979860**  **Genotype** | **Sustained Viral Response**  **(n=360)** | **No Sustained Viral Response**  **(n=434)** | **P value** | **OR, 95% CI** |
| --- | --- | --- | --- | --- | --- |
| **C1-C1** | CC | 60 (16.7) | 34 (7.8) | **1.25 x 10^-4^** | **0.43, 0.27 – 0.66** |
|  | CT | 67 (18.6) | 100 (23.0) | 0.13 |  |
|  | TT | 10 (2.8) | 33 (7.6) | **2.78 x 10^-3^** | **2.88, 1.40 – 5.93** |
| **C1-C2** | CC | 80 (22.2) | 43 (9.9) | **1.81 x 10^-6^** | **0.38, 0.26 – 0.57** |
|  | CT | 69 (19.2) | 100 (23.0) | 0.18 |  |
|  | TT | 24 (6.7) | 35 (8.1) | 0.45 |  |
| **C2-C2** | CC | 22 (6.1) | 13 (3.0) | **3.33 x 10^-2^** | **0.47, 0.24 – 0.96** |
|  | CT | 23 (6.4) | 60 (13.8) | **6.52 x 10^-4^** | **2.35, 1.42 – 3.89** |
|  | TT | 5 (1.4) | 16 (3.7) | **4.47 x 10^-2^** | **2.72, 0.99 – 7.49** |
|  |  |  |  |  |  |
| **C2C2** | T* | 28 (7.8) | 76 (17.5) | **5.18 x 10^-5^** | **2.52, 1.59 – 3.98** |
| **C1*** | CC | 140 (38.9) | 77 (17.7) | **2.80 x 10^-11^** | **0.34, 0.24-0.47** |
|  |  |  |  |  |  |
