# Supplementary material for: IL28B, HLA-C, and KIR Variants Additively Predict Response to Therapy in Chronic Hepatitis C Virus Infection in a European Cohort: A Cross-Sectional Study
Source: PLoS Med. 2011 Sep 13;8(9):e1001092. doi: 10.1371/journal.pmed.1001092 (PMC3172251; doi:10.1371/journal.pmed.1001092)
Supplement: Table S10 — Odds ratios and corresponding p-values for predicting failure of SVR using logistic regression models. (DOC) [file pmed.1001092.s012.doc]

**Table S10.** Odds ratios and corresponding p-values for predicting failure of SVR using logistic regression models

| **HLA C2C2/IL28B G***  **Two-way Interaction** | **OR** | **p value**  **(uncorrected)** |
| --- | --- | --- |
| **Intercept** |  | 0.03 |
| **HLA C2C2** |  | 0.56 |
| **IL28B G*** | 1.91 | 2.0x10^-5^ |
| **C2C2 and IL28B G*** | 5.05 | 0.04 |
|  |  |  |
| **HLA C2C2/IL28B G*/Not KIR2DS1**  **Three Way Interaction** |  |  |
| **Intercept** |  | 0.18 |
| **HLA C2C2** |  | 0.73 |
| **IL28B G*** | 2.04 | 6.29x10^-3^ |
| **Not KIR2DS1** |  | 0.72 |
| **C2C2 and IL28B G*** | 17.40 | 9.02x10^-3^ |
| **C2C2 and Not 2DS1** |  | 0.34 |
| **IL28B G* and Not 2DS1** |  | 0.75 |
| **C2C2 and IL28B G*and Not 2DS1** | 3.70 | 0.04 |
|  |  |  |
