# Supplementary material for: IL28B, HLA-C, and KIR Variants Additively Predict Response to Therapy in Chronic Hepatitis C Virus Infection in a European Cohort: A Cross-Sectional Study
Source: PLoS Med. 2011 Sep 13;8(9):e1001092. doi: 10.1371/journal.pmed.1001092 (PMC3172251; doi:10.1371/journal.pmed.1001092)
Supplement: Table S11 — The distribution of the six common IL28B haplotypes bound by SNPs rs12980275 and rs8099917. (DOC) [file pmed.1001092.s013.doc]

**Table S11.The distribution of the six common IL28B haplotypes bound by SNPs rs12980275 and rs8099917. The order is: rs12980275, rs12982533, rs8105790, rs688187, rs11881222, rs8103142, rs12979860, rs10853727, rs8109886, rs8099917.**

| No. | Haplotype | Frequency  (%) | Responders  (%) | Non-responders (%) | P value | OR^a^,  95% CI |
| --- | --- | --- | --- | --- | --- | --- |
| 1 | **ATTGATCTCT** | 43.2 | 48.4 | 39.6 | 4.00 x 10^-4^ | 0.70,  0.57 – 0.85 |
| 2 | **GCCAGCTTAG** | 23.8 | 16.5 | 30.4 | 9.51 x 10^-11^ | 2.20,  1.72 – 2.80 |
| 3 | **GCTAGCTCAT** | 10.3 | 10.1 | 10.6 | 0.77 | 1.04,  0.75 – 1.43 |
| 4 | **ATTGATCTAT** | 9.8 | 12.3 | 7.8 | 2.40 x 10^-3^ | 0.60,  0.43 – 0.83 |
| 5 | **ATTAACTTAT** | 1.9 | 2.2 | 1.7 | 0.47 | 0.79,  0.39 – 1.60 |
| 6 | **GCCAGCTTAT** | 1.4 | 1.1 | 1.6 | 0.39 | 1.49,  0.62 – 3.56 |

^a^ORs have been calculated as carriage of the haplotype vs non-carriage of the haplotype.
