# Supplementary material for: IL28B, HLA-C, and KIR Variants Additively Predict Response to Therapy in Chronic Hepatitis C Virus Infection in a European Cohort: A Cross-Sectional Study
Source: PLoS Med. 2011 Sep 13;8(9):e1001092. doi: 10.1371/journal.pmed.1001092 (PMC3172251; doi:10.1371/journal.pmed.1001092)
Supplement: Text S1 — Supplementary methods. (DOC) [file pmed.1001092.s014.doc]

**Text S1: Supplementary Methods**

**Genotyping**

For *HLA-C*, all samples (except Turin and spontaneous clearers) were genotyped by multiplex PCR to 2-digit resolution (1) with some modifications. Briefly, exon 2 and 3 of the HLA-C locus was amplified using the primers HLAC.M13F + HLAC15M13R (5' TGT AAA ACG ACG GCC AGT ARC GAG GKG CCC KCC CGG CGA 3' - 5' CAG GAA ACA GCT ATG ACC GGA GAT RGG GAA GGC TCC CCA CT 3') respectively. The resultant amplicon was sequenced using the primers M13F and M13R as well as the primers CIN2R (5' GGA GRC GTG ACC TGC GCC CCR GG 3'), CF1C( 5' CGG GGG CGG GGC CAG 3'), CX2RB (5' GCC CAG GAT CCG CAG GC 3'), CX2FG (5' GAG TGA ACC TGC GGA AA 3'), CX2FA (5' CAA CCA GAG CGA GGA CG 3') and CX2FD (5' ACC GGG AGA CAC AGA AG 3') to resolve likely ambiguities. Allele assignments were obtained using the program Assign (Conexio Genomics). In the large majority of cases, ambiguities were resolved following sequencing with allele-specific subtyping primers. However, in 5% of typings, identity at exons 2 and 3 was consistent with the presence of rare alternative and null alleles, and only in these rare cases was the allele assignment consistently allocated for the most common expressed allele and haplotype combination in the relevant population. Turin spontaneous clearers *HLA-C* genotyping was by PCR and sequencing using the following primer set: *HLA-C* forward primer: 5’-GGA GCC GCG CAG GGA-3’, *HLA-C* reverse primer: 5’-AGG GGT CGT GAC CTG CG-3’ and *HLA-C* sequencing primer: 5’-TAT TGG GAC CGG GAG ACA CAG-3’.

*KIR* genotyping. *KIR* genotyping was performed using previously described methods (2) with specific *KIR* group selections (see results).

IL28B rs12979860 SNP was genotyped using custom made taqman genotyping kit.

1. Witt, C. S., P. Price, G. Kaur, K. Cheong, U. Kanga, D. Sayer, F. Christiansen, and N. K. Mehra. 2002. Common HLA-B8-DR3 haplotype in Northern India is different from that found in Europe. Tissue Antigens 60:474-480.

2. Smita Kulkarni, Maureen P. Martin, and Mary Carrington. 2009. KIR Genotyping by Multiplex PCR-SSP. In Natural Killer Cell Protocols: Cellular and Molecular Methods. Series: Methods in Molecular Biology Volume No.: 612: 365-375.
